# Ultrahigh-Resolution NMR Spectroscopy**

**DOI:** 10.1002/anie.201404111

**Published:** 2014-05-26

**Authors:** Mohammadali Foroozandeh, Ralph W Adams, Nicola J Meharry, Damien Jeannerat, Mathias Nilsson, Gareth A Morris

**Affiliations:** School of Chemistry, University of ManchesterOxford Road, Manchester M13 9PL (UK)Homepage: http://nmr.chemistry.manchester.ac.uk; Department of Organic Chemistry, University of Geneva30 Quai E. Ansermet, 1211 Geneva 4 (Switzerland); Department of Food Science, University of CopenhagenRolighedsvej 30, 1958 Frederiksberg C (Denmark)

**Keywords:** chirp pulses, homonuclear decoupling, NMR spectroscopy, PSYCHE, structure elucidation

Spectral resolution is vital in NMR spectroscopy, but is instrument-limited. Recent “pure shift” pulse-sequence developments greatly improve resolution, but often at a high cost in sensitivity. We introduce a new class of pure shift experiments (PSYCHE) with superior sensitivity, spectral purity, and tolerance of strong coupling.

The key parameters for any spectroscopic technique are sensitivity and resolution. In the case of NMR spectroscopy, the sensitivity gains provided by the introduction of Fourier transform methods^1^ and of improved probe technologies make spectral resolution the limiting factor for most applications. From the early days of NMR spectroscopy it has been recognized that for some nuclei, in particular the proton, large gains in resolution could be achieved if the effects of homonuclear spin–spin couplings could be suppressed, but for many years all the methods proposed proved more or less unsatisfactory. Recently, much more practical methods, so-called “pure shift” or “chemical shift” methods have emerged, which partially or completely suppress the effects of homonuclear coupling, thereby generating spectra consisting of a single signal for each chemically distinct site, but generally at a high price in terms of sensitivity. Here we present a versatile new approach to pure shift NMR spectroscopy which has approximately tenfold better sensitivity than competing methods, gives clean spectra, and is tolerant of strong coupling.

In conventional proton NMR spectroscopy, scalar couplings (*J*) between protons carry structural information but reduce resolution, with the splitting of signals into multiplets greatly increasing signal overlap and complicating analysis and the assignment of spectra. The effects of heteronuclear couplings, for example between ^1^H and ^13^C, can easily be suppressed by using appropriate pulses during evolution times and broadband decoupling during detection. Homonuclear couplings are much more challenging, but the prize is a spectrum without multiplet structure, with only a single signal per chemical shift. In ^1^H NMR spectroscopy, this can represent a resolution improvement of almost an order of magnitude over conventional NMR spectroscopy; by way of comparison, 30 years of magnet development have delivered only a factor of two improvement, from 500 MHz to 1 GHz.

Recent developments have finally allowed such pure shift NMR spectra to be obtained, albeit at a significant cost in sensitivity. The elegant method of Zangger and Sterk (ZS)^2^ uses a frequency- and spatially selective 180° pulse; it has been enhanced and adapted for 1D NMR,^3^ DOSY,^4^ and 2D experiments such as TOCSY^5^ and NOESY.^6^ The ZS method is effective, but its sensitivity falls rapidly as the chemical shift difference between the resonances to be decoupled decreases.

The BIRD method^7^ relies on isotopically sparse heteronuclei. It typically selects protons directly bonded to ^13^C nuclei at natural abundance, so has a minimum sensitivity penalty of two orders of magnitude; it does not decouple geminal interactions, and suppresses signals of protons not bound to ^13^C nuclei. In experiments such as HSQC, however, which already rely on the presence of ^13^C nuclei, there is no additional sensitivity penalty and BIRD pure shift methods are highly effective.^8^ Both the ZS and BIRD methods are typically used to construct a pure shift interferogram, which can be Fourier transformed to yield a decoupled spectrum from a series of short chunks of data acquisition of duration 1/SW_1_;3a real-time windowed acquisition can sometimes be used to speed up experiments, but at some cost to spectral quality and resolution.^9^

**Figure 1:**
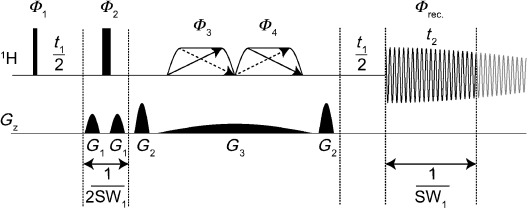
Pulse sequence for PSYCHE (pure shift yielded by chirp excitation). Narrow rectangles are 90° RF pulses, wide rectangles are 180° pulses, and trapezoids with diagonal arrows are both low-power frequency-swept chirp pulses of net flip angle *β*≪90°; optionally, pulses which sweep frequency in opposite directions simultaneously may be used, as indicated by the dotted arrows. *G*_1_, *G*_2_, and *G*_3_ are pulsed field gradients with half sine shapes. The highlighted part of the FID with duration 1/SW_1_ shows the chunk of data acquired for each increment of *t*_1_.

A new robust, general method is presented in Figure 1 that uses low flip angle (*β*) swept-frequency pulses in the presence of a weak magnetic field gradient. The method is related to the anti-z-COSY^10^, ^11^ experiment, but avoids the latter’s long minimum acquisition times, awkward data processing, and failure to deal efficiently with unwanted coherence transfer and strong coupling. The combined effect of the two *β* pulses is to refocus a small proportion of spins (the active spins) in a stimulated echo, while leaving the majority (the passive spins) unaffected. Taken together with the hard 180° pulse and the remaining field gradient pulses, the overall effect is to leave the net evolution of the active spins unchanged, but to invert the passive spins and to dephase all but the required single quantum coherences of the active spins. The distinction between passive and active spins is purely statistical, the former being a proportion sin^2^*β* of the whole and the latter cos^2^*β*, rather than being determined by spatial position within the sample (ZS method) or by coupling to a dilute heteronucleus (BIRD). As in previous experiments, data are acquired for a time 1/SW_1_, where SW_1_ is typically about twice the width of the widest multiplet to be decoupled, thus allowing a complete pure shift interferogram (equivalent to a free induction decay, FID) to be constructed in only a few dozen measurements.

The apparent simplicity of the “double *β*” element hides some subtleties. If hard low flip angle pulses are used, as in the anti-z-COSY experiment, a number of unwanted coherence transfer pathways survive, in particular those (“cross-peak” pathways^11^) in which coherence is transferred between coupled spins by the pair of pulses, and those involving zero quantum coherence (ZQC) in the interval between the pulses.^12^ The combination of frequency-swept pulses and field gradient effectively superposes signals with a range of different ZQC evolution times from different sample regions, thereby suppressing the effect of ZQCs (as in Keeler’s zero quantum suppression method^12^). The coherence transfer pathway required is one in which the coherence order is opposite on either side of the chirp pulse pair, and zero between the two pulses. This means that cross-peak pathways are suppressed, because for these the two frequency sweeps are on resonance at different times, so that the gradient pulse areas experienced before the initial coherence is converted into *z*-magnetization and after the magnetization is converted back into coherence are different.

In the suppression of both zero quantum and cross-peak pathways, the lower limit of the chemical shift difference for which the suppression is effective is governed by the frequency sweep rate and gradient amplitude. Coherence transfer pathways that originate from strong coupling^13^ are also attenuated.

The principal remaining sources of unwanted signals are coherence pathways in which each *β* pulse affects two spins at once. While the desired pure shift signals have amplitudes proportional to sin^2^*β*, the unwanted pathways, which give rise to weak spurious signals, have amplitudes proportional to sin^4^*β* (see Figure S6 in the Supporting Information). This means that, uniquely among current pure shift methods, PSYCHE allows the experimenter to control the compromise between sensitivity and spectral purity, by adjusting the value of *β* to give the maximum signal-to-noise ratio compatible with the required degree of freedom from spectral artefacts. A further improvement (by a factor ${\sqrt 2 {$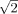) can be obtained by using a double frequency sweep, as indicated by the dotted arrows in Figure 1. In critical cases, *β* can be chosen so that the artefact level is just below that of the noise, thereby maximizing the sensitivity without sacrificing spectral quality. The main class of pure shift methods that can currently compete with PSYCHE in terms of sensitivity is that of band-selective methods.^9^, ^14^ Even though these decouple only part of the spectrum, they have already found application in biomolecular NMR spectroscopy.^14^ Provided that the natural line broadening allows a gain in resolution by decoupling, the use of PSYCHE in this field is attractive for both homo- and heteronuclear experiments, as broadband homodecoupling is achieved.

**Figure 2:**
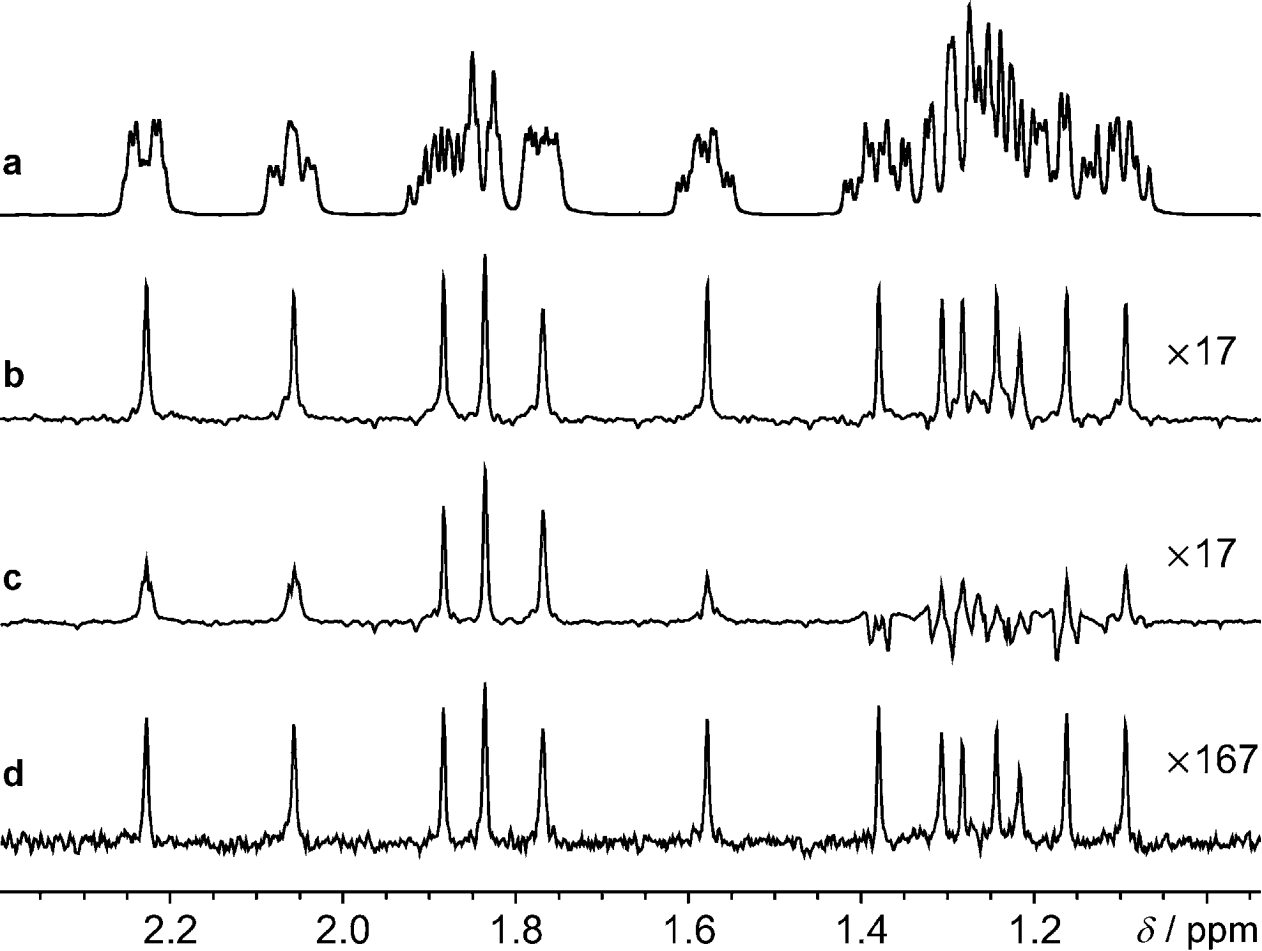
Spectra obtained by normal ^1^H NMR spectroscopy (a), PSYCHE (b), ZS using a 12 ms rsnob refocusing pulse (c), and ZS using a 100 ms rsnob refocusing pulse (d) of a sample of estradiol in [D_6_]DMSO. Spectrum (a) and each of spectra (b), (c), and (d) were acquired in experiment times of 15 s and 6 min, respectively; full experimental details are given in the Supporting Information.

**Figure 3:**
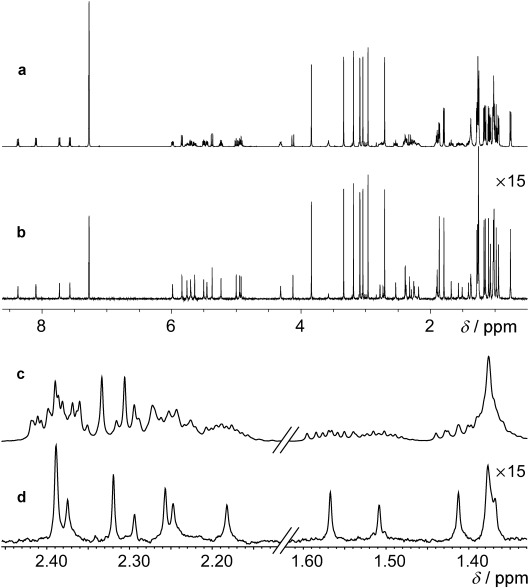
Normal ^1^H NMR (a) and PSYCHE (b) spectra of a sample of cyclosporine A in [D_6_]benzene, with corresponding expansions (c) and (d). Spectra (a) and (b) were acquired in experiment times of 15 s and 6 min, respectively; full experimental details are given in the Supporting Information.

Figure 2 illustrates the application of the PSYCHE and ZS pure shift methods to the challenging case of estradiol, which has a crowded spectrum with significant strong coupling (Figure 2 a). Figure 2 b shows the result of PSYCHE, with close to perfect decoupling and a single signal for each distinct chemical shift, and Figure 2 c that of a ZS experiment with parameters chosen to give the same sensitivity. Decoupling fails for almost all the signals in the latter; only by using a highly selective pulse, at a cost of a factor of 10 in sensitivity (Figure 2 d) does the ZS method approach the same quality of decoupling. The results with a BIRD sequence would be similar to those of Figure 2 d, but with doublets for geminal protons, since BIRD is unable to decouple protons attached to the same ^13^C nucleus. Figure 3 illustrates the application of PSYCHE to a cyclic peptide (cyclosporin A), and shows a significant enhancement in spectral resolution. Further examples for a small molecule (azithromycin) and a protein (ubiquitin) are given in the Supporting Information, along with instructions for generating the chirp pulses and pulse sequence codes.

The PSYCHE method is flexible, general, and offers almost an order of magnitude improvement in performance over existing methods for broadband homonuclear decoupling. It has the potential to find wide application in the NMR spectroscopy of small molecules, NMR-based metabolomics, and biomolecular NMR spectroscopy.
