# Supplementary material for: Ultrahigh-Resolution NMR Spectroscopy
Source: Angew Chem Int Ed Engl. 2014 May 26;53(27):6990–2. doi: 10.1002/anie.201404111 (PMC4320760; doi:10.1002/anie.201404111)
Supplement: Supplementary file 1 [file anie0053-6990-sd1.pdf]

Angewandte Chemie
Eine Zeitschrift der Gesellschaft Deutscher Chemiker

Supporting Information

© Wiley-VCH 2014

69451 Weinheim, Germany

Ultrahigh-Resolution NMR Spectroscopy**

*Mohammadali Foroozandeh, Ralph W. Adams, Nicola J. Meharry, Damien Jeannerat, Mathias Nilsson, and Gareth A. Morris**

anie_201404111_sm_miscellaneous_information.pdf

Experimental section

All spectra were recorded at 298 K on a Bruker Avance II+ 500 MHz spectrometer with a 5 mm BBO probe equipped with a z-gradient coil with a maximum nominal gradient strength of 53 G cm^{-1}. The durations of the 90-degree pulse was 11.6 µs for ^{1}H. The spectral windows were set to 5 kHz (10 ppm). The carrier frequencies were set to 5 ppm. The chirp pulses were centred at 5 ppm and their durations were set to 15 ms each, to cover about 6 kHz with an amplitude of 46 Hz and a flip angle β of 20°. G_1 and G_2 gradients are used for selection of desired coherence transfer pathways with amplitudes of 24 G cm^{-1} and 38 G cm^{-1} respectively and duration of 1.5 ms each. The G_3 gradient pulse was aligned with the centre of the pulse element consisting of two chirp pulses with an amplitude of 0.75 G cm^{-1}. All gradient pulses were followed by a recovery delay of 1.5 ms. The spectral windows in F_1 and F_2 were set to 40 Hz and 5 kHz respectively. The number of t_1 increments (number of chunks) and number of scans were set to 32 and 4, respectively, for all pure shift spectra, with a total experiment time of 6 minutes for each. Φ_1=*x*,-*x*, Φ_2=*x*, Φ_3=Φ_4=*x*,*x*,-*x*,-*x* and $\Phi_{rec.}$=*x*,-*x*.

Generation of Chirp pulses

Chirp pulse elements used in the PSYCHE pulse sequence were generated and integrated in the Shape Tool of Topspin software version 2.1. The two chirp pulses in the double chirp element were generated with the following parameters:

Size of Shape: 10000

Total Sweep-Width: 10000 Hz

Length of Pulse: 15000 uses

% to be smoothed: 20 %

Q (for the middle of shape): 5.0

For the low-to-high pulse, the first optional box (Low to High Field) was checked, while for the high-to-low pulse it was unchecked. Pulse elements Low-to-High/ High-to-Low and High-to-Low/Low-to-High were generated by concatenating the text files of individual pulses. The mixed pulse, which sweeps frequencies from low-to-high and high-to-low field simultaneously, was generated by using the Add Shape option in the Manipulate menu of Shape Tool, or by the command "manipul addshape", aligning at the centre and rescaling the amplitude to 100 %.

Calibration of Chirp pulses with small flip angle

The Chirp pulses used may be generated by using the spectrometer software to calculate an adiabatic inversion (180°) pulse and then reducing the radiofrequency (RF) power. To find the RF field strength ($\gamma B_1/2\pi$) needed to generate a PSYCHE chirp pulse element of flip angle β, the RF field ($\gamma B_{1(\max)}/2\pi$) for a 180° rotation is first calculated by integrating the pulse as an adiabatic shape via the Analysis menu of Bruker Shape Tool, or by using the command "analyze integradia". For the pulse used here, with a duration, τ_p, of 15 ms, a sweep-width, ΔF, of 10 kHz, and an adiabaticity factor, Q, of 5, an RF field of 728 Hz is obtained for $\gamma B_{1(\max)}/2\pi$. $\gamma B_{1(\max)}/2\pi$ can also be calculated using **Equation S1**:

$$\frac{\gamma B_{1(\max)}}{2\pi} = \frac{\sqrt{\left(\tau_p \times \Delta F \times Q/2\pi\right)}}{\tau_p}$$ **Equation S1**

The relationship between flip angle and RF field strength for a chirp pulse of this shape but different amplitudes is illustrated in **Figure S9**. As is shown in **Figure S9b**, there is an approximately linear relationship between the desired flip angle and the RF amplitude of the chirp pulse for flip angles in the range 0° – 45°. For a given flip angle, the RF field strength required with the pulse shape described is given by **Equation S2**. For example, the PSYCHE chirp elements used in this work, with a flip angle of 20°, were obtained by reducing the RF field used for a chirp 180° pulse by a factor of 16, corresponding to an addition of 24 dB to the power attenuation of the shaped pulse (parameter SP11 of the pulse sequence psyche.mf in the **Supplementary Software** below).

$$\frac{\gamma B_{1(\beta)}}{2\pi} = 2.27 \times \beta$$ **Equation S2**

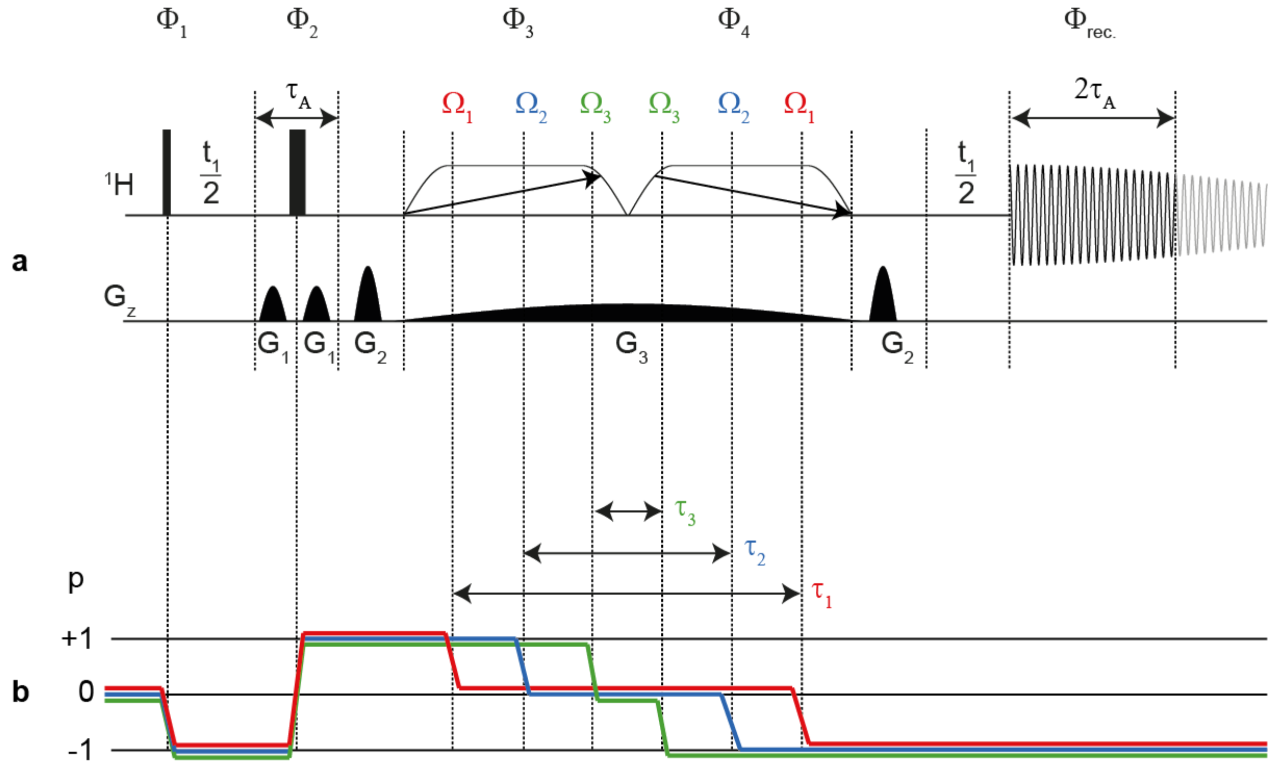

Figure S1. a) Pulse sequence for PSYCHE, Pure Shift Yielded by Chirp Excitation. Narrow rectangles are 90° RF pulses, wide are 180° pulses, and trapezoids with diagonal arrows are low-power chirp pulses which sweep frequency in opposite directions. τ_A=1/2SW1, where SW1 is the spectral window in F_1 and **b)** Coherence transfer pathway diagram for three different resonance offsets, Ω_1, Ω_2 and Ω_3 during the pulse sequence.

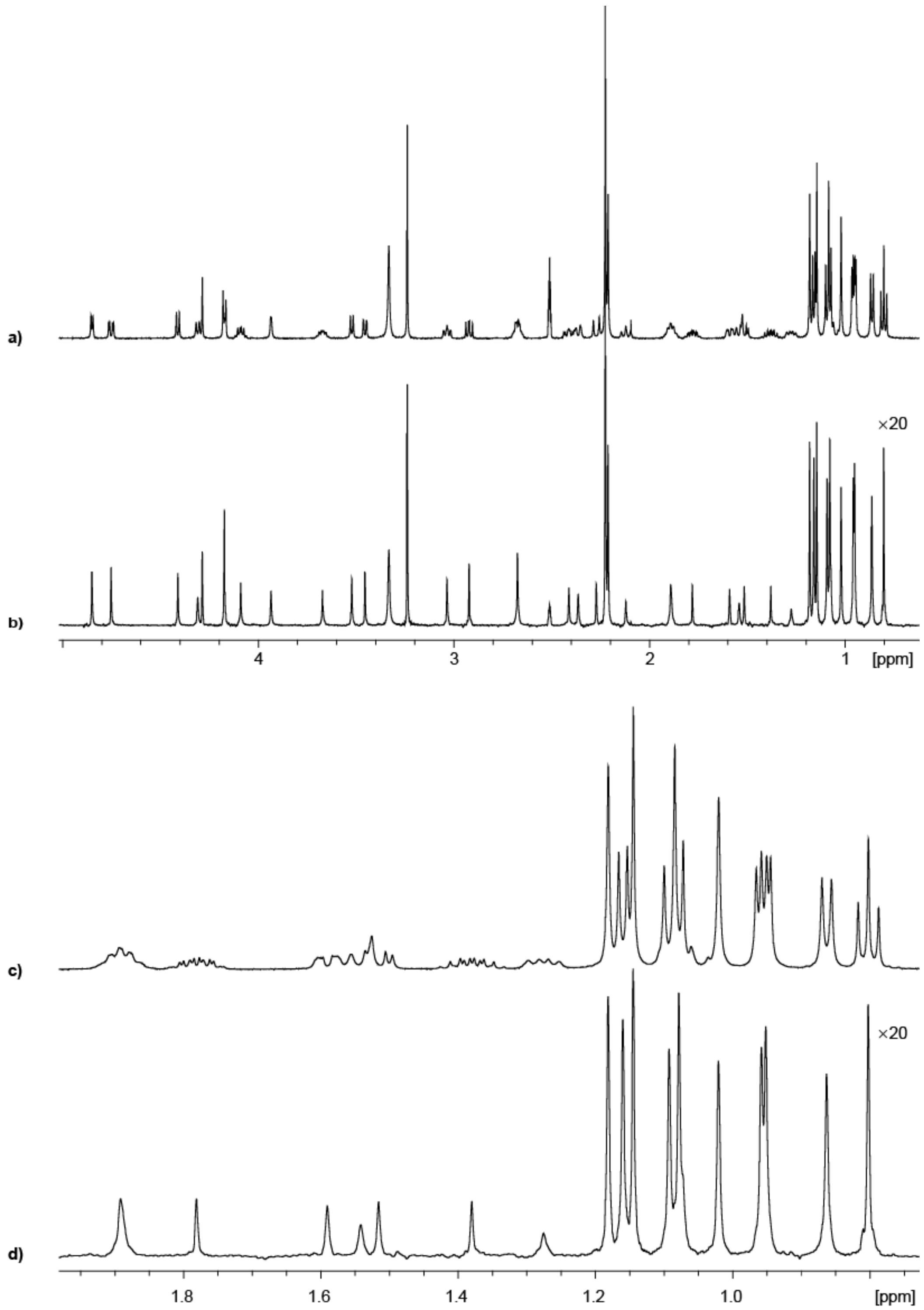

Figure S2. Normal ^{1}H NMR (**a**) and PSYCHE (**b**) spectra of a 72 mM sample of azithromycin in DMSO-d_6, with expansions (**c**) and (**d**) respectively.

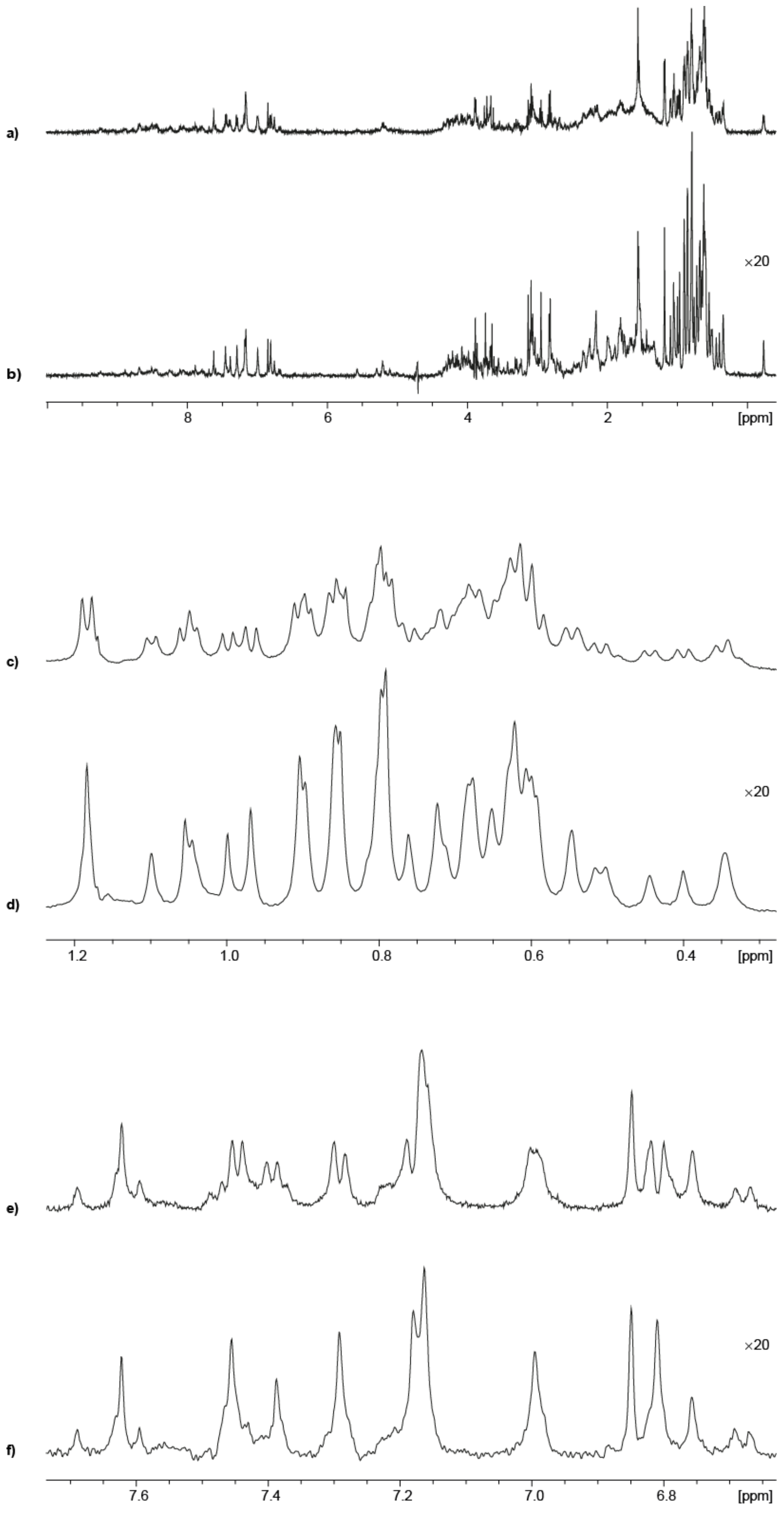

Figure S3. Normal ^{1}H NMR (**a**) and PSYCHE (**b**) spectra of a 1 mM sample of ubiquitin in 90% H_2O/10% D_2O, with corresponding expansions (**c**) to (**f**). Spectrum **b** was obtained by adding water suppression using excitation sculpting to the PSYCHE sequence (**Figure S4**).

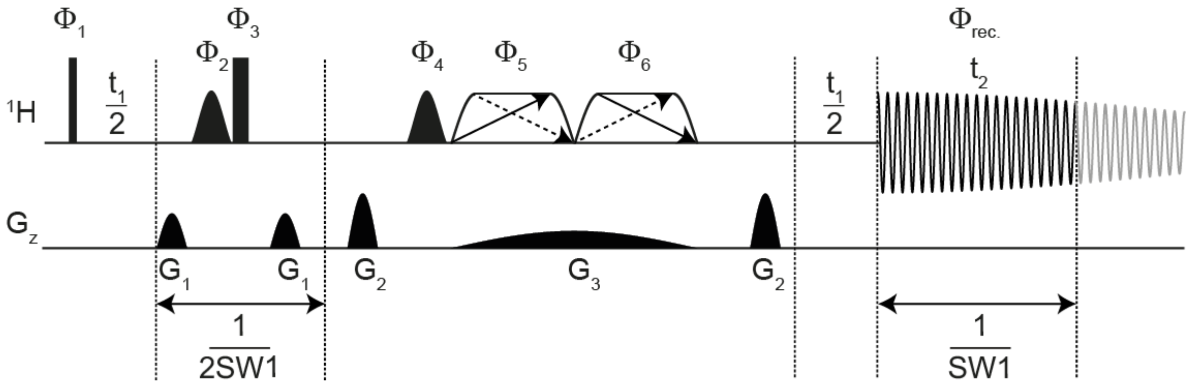

Figure S4. Pulse sequence for PSYCHE with water suppression using excitation sculpting. Narrow rectangles are 90° RF pulses, wide are 180° pulses, and trapezoids with cross-diagonal arrows are low-power chirp pulses, with a flip angle of 20°, which sweep frequency in opposite directions simultaneously and have a duration of 15 ms and an amplitude of 46 Hz. Shaped pulses are 180° sinc pulses of duration 3 ms and amplitude 283 Hz, for selective water inversion. G_1, G_2 and G_3 are pulsed field gradients with half sine shapes. The G_1 and G_2 gradient pulses are used for selection of the desired coherence transfer pathways (CTP) and have amplitudes of 24 G cm^{-1} and 38 G cm^{-1} respectively and a duration of 1.5 ms each. The G_3 gradient pulse is aligned with the midpoint of the two chirp pulses and has an amplitude of 0.75 G cm^{-1}. Φ_1=*x*,-*x*, Φ_2=-*x*,-*x*,-*x*,-*x*,*x*,*x*,*x*,*x*, Φ_3=*x*, *x*, *x*, *x*, -*x*,-*x*,-*x*,-*x*, Φ_4=-*x*,-*x*,-*y*,-*y*, Φ_5=Φ_6=*x*,*x*,*y*,*y* and $\Phi_{rec.}$=*x*,-*x*,-*x*,*x*,-*x*,*x*,*x*,-*x*.

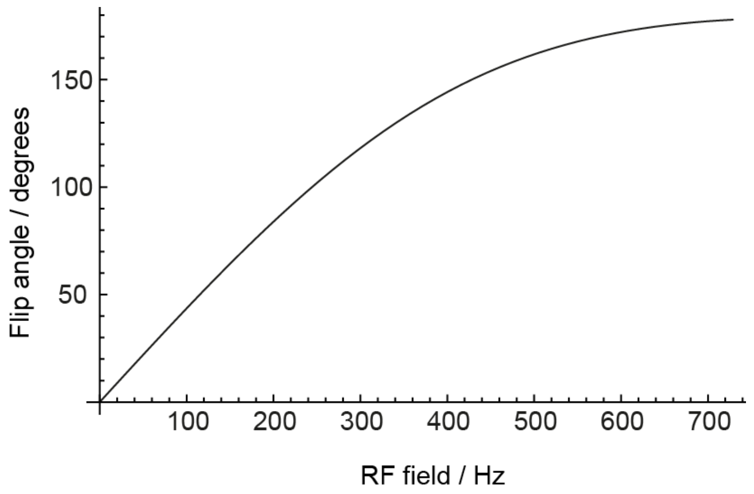

Figure S5. Graphs illustrating the relationship between the flip angle (in degrees) and the RF field (in Hz) of the shaped pulse used in the PSYCHE chirp pulse element.

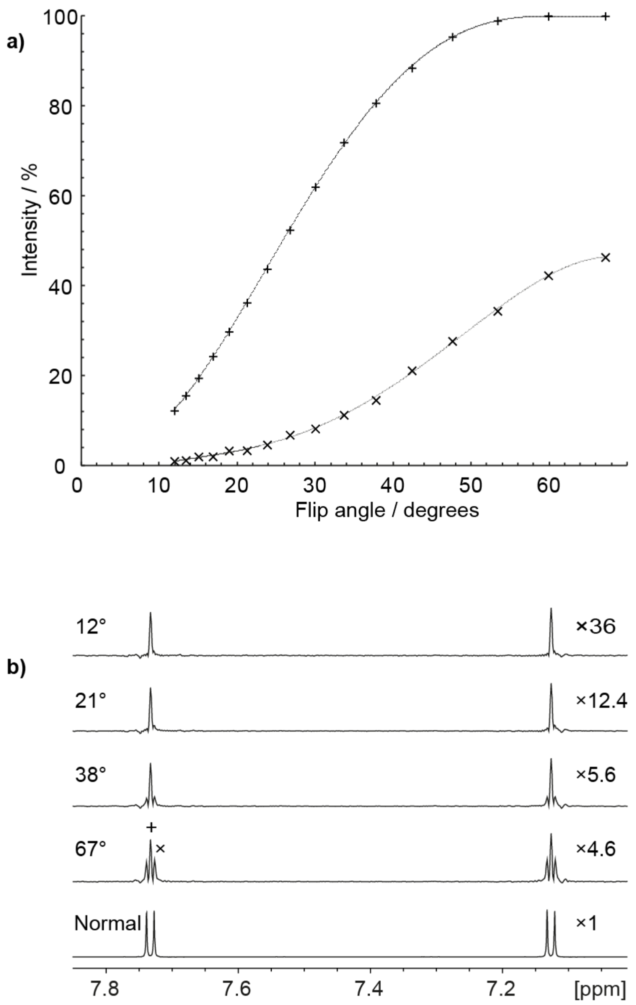

Figure S6. (**a**) Graph showing the relative experimental intensities of the pure shift signals (+) and the recoupling artifacts (×) as a function of β, and (**b**) corresponding experimental spectra, with flip angles β and vertical expansion factors marked, for the AX spin system of 2,3-dibromothiophene in DMSO-d_6 .

Pulse sequence codes

```
; psyche.mf
;
; Pure Shift Yielded by Chirp Excitation
; J is refocused at centre of chunk
;
; Mohammadali Foroozandeh
; University of Manchester
; (26/11/2013)
; Avance II+/III Version
;
; Data can be reconstructed using a macro available at http://nmr.chemistry.manchester.ac.uk

;$CLASS=HighRes
;$DIM=2D
;$TYPE=
;$SUBTYPE=
;$COMMENT=

#include <Avance.incl>
#include <Grad.incl>

define delay tauA
define delay tauB

"in0=inf1/2"
"p2=p1*2"
"p12=p11+d18"
"tauA=in0/2-p16-d16-50u"
"tauB=(dw*2*cnst4)+d16+50u"

1   ze
2   d1 pl1:f1
3   p1 ph1
    d0
    tauA
    50u UNBLKGRAD
    p16:gp1
    d16
    p2 ph2
    50u
    p16:gp1
    d16
    tauA
    p17:gp2
    d17
    10u pl0:f1
    tauB
    ( center (p12:gp12) (p11:sp11 ph3):f1 )
    d16
    10u pl1:f1
    p17:gp2
    d17
    50u BLKGRAD
    d0
 4  go=2 ph31
    d1 mc #0 to 2 F1QF(id0)
exit

;Phase Cycling

;ph1                ; Hard_90
;ph2                ; Hard_180
;ph3                ; Selective_180
;ph31               ; Receiver

ph1= 0 2
ph2= 0
ph3= 0 0 2 2
ph31=0 2

;p1: high power 90 pulse width
;p2: high power 180 pulse width
;p11: duration of PSYCHE chirp pulse element
```

```
;p12: duration of gradient during PSYCHE chirp pulse element

;p16: CTP gradient pulse width
;p17: CTP gradient pulse width
;pl1: f1 channel - power level for pulse (default)
;pl10: 120 dB

;sp12: power level of PSYCHE chirp element
;spoffs12: selective pulse offset (0 Hz)
;spnam12:  file name for selective pulse

;gpz1: CTP gradient 49%
;gpz2: CTP gradient 77%
;gpz12: gradient 1-3 %
;gpnam1: SINE.100
;gpnam2: SINE.100
;gpnam12: SINE.100

;d0: incremented delay, set initial value to 0 s
;d1: relaxation delay; 1-5 * T1
;d16: gradient stabilisation delay
;d17: gradient stabilisation delay
;td1: number of chunks to acquire

;NS: number of scans
;DS: number of dummy scans
;td1: number of experiments
;cnst4: number of points to drop when collecting FID
;
;FnMODE: QF

%%%%%%%%%%%%%%%%%%%%%%%%%%%%%%%%%%%%%%%%%%%%%%%%%%%%%%%%%%%%%%%%%%%%%%%

; psychees.mf
;
; Pure shift yielded by chirp excitation
; J is refocussed at centre of chunk
; With water suppression using ecxitation sculpting
;
; Mohammadali Foroozandeh
; University of Manchester
; (10/2/2014)
; Avance III Version
;
;
; Data can be reconstructed using the 'pshift' macro.

;$CLASS=HighRes
;$DIM=2D
;$TYPE=
;$SUBTYPE=
;$COMMENT=

#include <Avance.incl>
#include <Grad.incl>

define delay tauA
define delay tauB

"in0=inf1/2"
"p2=p1*2"
"p12=p11+d18"
"tauA=in0/2-p16-d16-p13-50u-10u-4u"
"tauB=(dw*2*cnst4)+d16+50u"

1 ze
2 d1 pl1:f1
3 p1 ph1                ; 90
  d0                    ; Incremented delay
  tauA
  50u UNBLKGRAD
  p16:gp1
  d16 pl0:f1
  (p13:sp1 ph2:r):f1
  4u
```

```
  10u pl1:f1
  (p2 ph3)
  4u
  10u pl0:f1
  50u
  p16:gp1
  d16
  tauA
  p17:gp2
  d17
  tauB
  (p13:sp1 ph4:r):f1
  10u
  (center (p12:gp12) (p11:sp11 ph5):f1 )
  d16
  10u pl1:f1
  p17:gp2
  d17
  50u BLKGRAD
  d0                        ; incremented delay
4 go=2 ph31
  d1 mc #0 to 2 F1QF(id0)

exit

ph1=  0 2 0 2 0 2 0 2
ph2=  2 2 2 2 3 3 3 3
ph3=  0 0 0 0 1 1 1 1
ph4=  2 2 3 3 2 2 3 3
ph5=  0 0 1 1 0 0 1 1
ph31=0 2 2 0 2 0 0 2

;ph31= ph1 + ph3*2 + ph5*2                  ; Receiver

;p1: high power 90 pulse width
;p2: high power 180 pulse width
;p11: duration of PSYCHE chirp pulse element
;p12: duration of gradient during PSYCHE chirp pulse element
;p13: duration of water suppression shaped pulse

;p16: CTP gradient pulse width
;p17: CTP gradient pulse width
;pl1: f1 channel - power level for pulse (default)
;pl10: 120 dB

;sp1: power level of water suppression shaped pulse
;spoffs1: selective pulse offset (0 Hz)
;spnam1:  file name for selective pulse

;sp11: power level of PSYCHE chirp element
;spoffs11: selective pulse offset (0 Hz)
;spnam11:  file name for selective pulse

;gpz1: CTP gradient 49%
;gpz2: CTP gradient 77%
;gpz12: gradient 1-3 %
;gpnam1: SINE.100
;gpnam2: SINE.100
;gpnam12: SINE.100

;d0: incremented delay, set initial value to 0 s
;d1: relaxation delay; 1-5 * T1
;d16: gradient stabilisation delay
;d17: gradient stabilisation delay
;td1: number of chunks to acquire

;NS: number of scans
;DS: number of dummy scans
;td1: number of experiments
;cnst4: number of points to drop when collecting FID
;
;FnMODE: QF
```
